# Tick-Borne Encephalitis (TBE): From Tick to Pathology

**DOI:** 10.3390/jcm12216859

**Published:** 2023-10-30

**Authors:** Dominic Adam Worku

**Affiliations:** 1 Infectious Diseases, Morriston Hospital, Heol Maes Eglwys, Morriston, Swansea SA6 6NL, UK; dominicworku@wales.nhs.uk; 2 Public Health Wales, 2 Capital Quarter, Cardiff CF10 4BZ, UK

**Keywords:** TBE, encephalitis, orthoflaviviruses, neurovirulence, diagnosis, presentation, management

## Abstract

Tick-borne encephalitis (TBE) is a viral arthropod infection, endemic to large parts of Europe and Asia, and is characterised by neurological involvement, which can range from mild to severe, and in 33–60% of cases, it leads to a post-encephalitis syndrome and long-term morbidity. While TBE virus, now identified as *Orthoflavivirus encephalitidis*, was originally isolated in 1937, the pathogenesis of TBE is not fully appreciated with the mode of transmission (blood, tick, alimentary), viral strain, host immune response, and age, likely helping to shape the disease phenotype that we explore in this review. Importantly, the incidence of TBE is increasing, and due to global warming, its epidemiology is evolving, with new foci of transmission reported across Europe and in the UK. As such, a better understanding of the symptomatology, diagnostics, treatment, and prevention of TBE is required to inform healthcare professionals going forward, which this review addresses in detail. To this end, the need for robust national surveillance data and randomised control trial data regarding the use of various antivirals (e.g., Galidesivir and 7-deaza-2′-CMA), monoclonal antibodies, and glucocorticoids is required to improve the management and outcomes of TBE.

## 1. Introduction

Tick-borne encephalitis (TBE) was first clinically described in the 18th century, with its causative agent tick-borne encephalitis virus (TBEV) now identified as *Orthoflavivirus encephalitidis*, first isolated in 1937 [1,2]. TBEV is transmitted primarily by infected hard ticks, such as *Ixodes ricinus* and *Ixodes persulcatus*, although other tick species may also act as a host, with transmission occurring within minutes of a tick bite [1]. TBEV is an orthoflavivirus, and as such, it is related to *Orthoflavivirus japonicum* (Japanese encephalitis, JE), *Orthoflavivirus nilense* (West Nile Virus, WNV), and *Orthoflavivirus louisense* (St. Louis encephalitis, SLE) and thus possesses a single-stranded positive-sense RNA (ssRNA) genome [2,3]. The control of orthoflaviviruses such as TBEV is challenging due to the presence of multiple animal reservoir hosts, which help to sustain the disease in areas of endemicity. Moreover, within humans, the majority of *Orthoflavivirus* infections are clinically inapparent (1:100, apparent: inapparent) making the identification of transmission sites challenging [3]. Where symptoms occur, however, there is a risk of central nervous system (CNS) involvement manifesting as encephalitis/meningitis/myelopathy, with the risks of long-term neuropsychiatric sequelae present in survivors [4,5,6]. 

Current data suggest that >10,000 TBE cases occur in Europe and Asia each year [1]. In 2019, 3411 TBE cases were reported in Europe, with a case fatality ratio of 0.7%. Moreover, TBE diagnoses in Europe doubled between 2015 and 2020, with Germany, Norway, Slovenia, and Switzerland responsible for this increase. However, these data are likely to be an underestimate, given that, in many European countries, mandatory reporting of TBE is not practiced [7,8,9,10]. It is estimated that, in TBE endemic areas, TBE may be responsible for approximately 10% of paediatric encephalitis cases [11]. 

**Figure 1:**
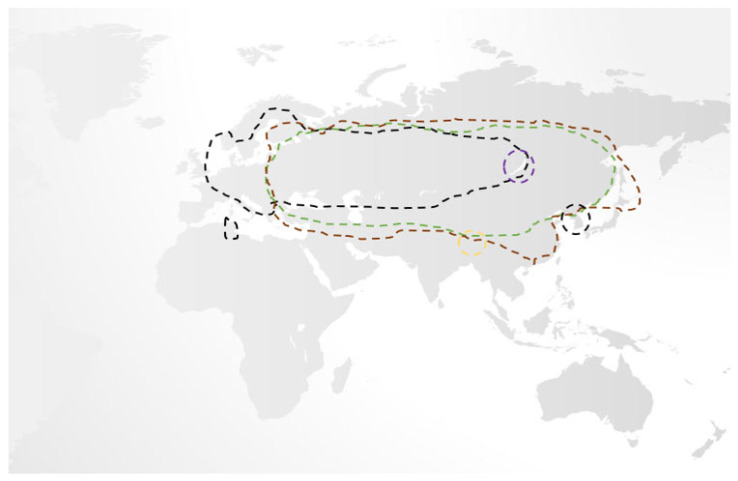
An overview of the geographical distribution of the five recognised subtypes of TBEV, which mirrors associated tick populations. Of these, European (black line), Siberian (brown line), and Far-Eastern (green line) are responsible for the majority of cases and have significant overlap, while the Baikalian (purple line) and Himalayan (gold line) are more geographically restricted [5,13,14,16,17,18,19].

While, traditionally, only three subtypes of TBEV were recognised, namely the European, Siberian, and Far-Eastern subtypes (each separated by 5–6% variation in their amino acid sequences with multiple variants therein), recent phylogenetic studies have identified two further subtypes, Himalayan and Baikalian. Unlike the former three, Himalayan and Baikalian subtypes are geographically restricted, are less studied, but have been isolated in human cases of TBE where they may be associated with a severe disease phenotype [12,13,14,15,16]. While European TBEV is primarily spread by the *Ixodes ricinus* tick, other hard ticks, notably *Ixodes persculatus* and *Dermacentor reticulatus*, may also do so. In contrast, Far-Eastern and Siberian TBEV is associated with the *Ixodes persulcatus* and *Ixodes ovatus* ticks. Unsurprisingly, there is remarkable geographical overlap amongst TBEV subtypes, making their differentiation challenging [5,13,14,16,17,18,19]. (Figure 1) Given that *Ixodes ricinus* and *Ixodes persulcatus* ticks may serve as the vector for several diseases, the possibility of coinfection should be considered [20,21].

Overall, the European subtype of TBEV is the most widespread, having been reported as far as Tunisia and South Korea, and uniquely, it may present in a biphasic manner. While the associated mortality of European TBEV is low (0.5–2% case fatality ratio) vs. the Far-Eastern/Siberian subtypes (20–40% case fatality ratio), it is more often associated with long-term sequelae. Overall, 5% of survivors will suffer permanent paresis, and approximately 33–60% will suffer a post-encephalitic syndrome that may last for years after TBE recovery [5,18,19,22,23]. 

While TBE has similarities to other tick-borne diseases, it has some important differences. As is the case with anaplasmosis and babeosis, TBEV transmission has been associated with blood transfusion and organ transplantation, highlighting the need for suitable screening of individuals heralding from endemic areas [24,25,26]. Uniquely, however, TBE has been associated with alimentary transmission from the ingestion of unpasteurised milk or cheese from goats, sheep, and cows from several areas of Europe (e.g., Slovakia, Czech Republic, Poland, Russia, Slovenia) and Asia, which has led to several important disease outbreaks [27]. As such, awareness of this method of transmission is of considerable importance in the setting of familial outbreaks of TBE and in returning travelers. Unlike other orthoflaviviruses, there is no evidence of the transmission of TBEV from mother to child or congenital malformation risk [28].

Therefore, TBE is a growing concern given the multiple transmission route, the ubiquity of the tick vector, and the increase in TBE incidence over the last 30 years despite the presence of an effective vaccine [29,30]. Pavlovsky was the first to demonstrate the involvement of ticks in the transmission of disease to humans and, in particular, TBEV. His natural focus theory focused on the determinants and non-human influences, which allow for pathogen persistence in a given ecosystem [31]. In his theory, three critical factors were identified, namely the vector, reservoir vertebrate host, and susceptible other hosts (e.g., humans), with contribution from relevant soil ecosystems [32]. While, in TBEV, the *Ixodes* tick is the main vector, with evidence that TBEV has coevolved to preferentially infect *Ixodes* ticks and to alter their behaviour (i.e., increased time questing), rodents (e.g., voles) are key reservoir hosts that assist in maintaining viraemic transmission [33,34,35]. While rodents were considered the chief mechanism by which tick population infection is maintained, it is clear that non-viraemic routes of transmission, including tick transovarial/transstadial transmission and transmission amongst co-feeding infected and non-infected ticks, are important contributors to TBEV persistence at these natural focuses given the fluctuations in reservoir host populations [33,35,36].

When we consider the ideal environment for tick survival and development, they include areas with stable temperatures of approximately 5–7 °C with high relative humidity (>80%) and include wetland forests, shrubbery and overgrown meadows [35,36,37]. These foci may range from metres to kilometers in size, making them challenging to identify, and thus, they serve as a self-sustaining environment for disease transmission [38,39]. 

The increase in TBE cases is likely multifactorial but almost certainly reflects the effects of climate change and land usage by humans (i.e., urbanization), which has helped create and sustain environments outside of endemic areas for tick populations e.g., *I.persulcatus* to thrive and remain active outside the typical months of March–July [37,40]. While it would seem that specific ecological, environmental, and geological factors are present in TBEV microfoci, these remain poorly understood. Current models do not explain recent temporal patterns of disease incidence in Europe and must be improved upon to assist in TBEV disease modelling and control methods [41]. In 2019, the United Kingdom (UK) described its first human case of TBEV, with native deer seroprevalence studies highlighting high rates of TBEV infection present particularly within the Thetford area of England, which has led to concern [42,43]. 

In this review, we explore key concepts of European TBE, such as its viral structure, the phases of the disease, and its diverse symptomatology. In doing so, we discuss host characteristics and the influence of the host immune response and how this influences the disease phenotype in the hopes of identifying potential biomarkers. Given that approximately 60% of viral encephalitis cases are managed without a diagnosis, this understanding is of extreme importance [44]. To conclude, we explore current management options and their respective evidence base and explore novel therapeutics being trialed in this arena, which are urgently required in order to improve TBE outcomes. 

## 2. TBEV Structure

**Figure 2:**
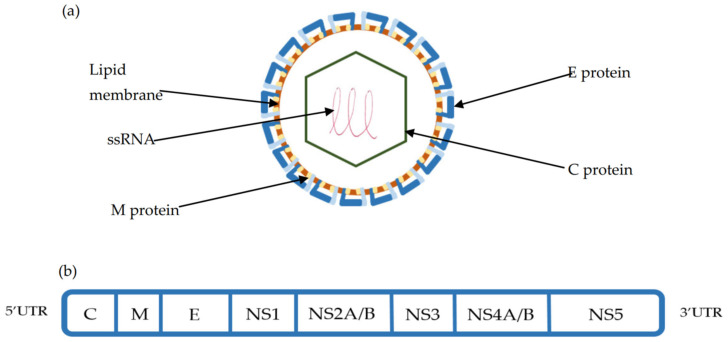
(**a**) TBEV viral structure composed of the Capsid (C), Membrane (M), and Envelope (E) proteins. The E protein is integral to cellular binding and is the target of the host humoral response. (**b**) The structure of the single-stranded RNA (ssRNA) open reading frame (ORF) is flanked by 3′ and 5′ untranslated regions. This ORF is cleaved using viral and host proteases into 3 structural (C, M, E) and 7 non-structural proteins (NS1-NS5) [45,46,47].

TBEV is a 50 nm enveloped Orthoflavivirus with an ssRNA 11kb genome, consisting of one open reading frame (ORF) encoding for 10 proteins. The polyprotein derived from the ORF is cleaved by both viral and cellular proteases into three structural (Capsid (C), premembrane (preM), Envelope (E) protein) and seven non-structural proteins (NS1,NS2a-b, NS3, NS4a-b, NS5), which help define the viral life cycle [45,46,47]. (Figure 2). With the use of cryo-electron micrography, the TBE viral structure has been fully appreciated. The icosahedral surface of TBEV is similar to other orthoflaviviruses and is made of the E protein that is associated both with attached surface glycan’s and the underlying M protein. The E and M proteins, which are embedded into the virus membrane and distort its shape, exist as a heterotetramer, with the E protein dimerised in a head to tail orientation. While the E protein consists of four domains, of which domain II is key for endosomal fusion and domain III is a key target for the neutralising antibody, the M protein consists of an N-terminal loop and perimembrane and transmembrane helices, which stabilize the E protein and prevent conformational change [47]. Underlying the surface is the C-protein, which exists in continuity with the ssRNA genome to form the nucleocapsid. The ssRNA genome is flanked by 3′ and 5′ untranslated regions (UTR), which are important regulators of TBEV genome replication, translation, and TBEV RNA migration in neurons [48,49].

The E protein is fundamental in TBEV cellular entry, which utilises receptor-mediated endocytosis and is the main target of the immune response [48]. Indeed, domain III of the E protein has been shown to be fundamental for TBEV neurovirulence, with mutations here drastically reducing the development of neurological disease in mice [49]. When compared to other orthoflaviviruses, including dengue virus, the E protein of TBEV shares <40% sequence homology, making it an obvious pharmacological target [50]. While no definitive cellular receptor has been identified for TBEV cellular entry, it is believed to be a multistep process. This includes initial low affinity binding through heparan sulphate proteoglycans, followed by high affinity interactions dependent on the underlying cell type, including laminin binding protein, α_v_β_3_ integrin, and T-cell immunoglobulin and mucin domain 1 (TIM-1) [51,52,53].

Upon cellular entry, TBEV attenuates cellular processes with structural changes noted to the endoplasmic reticulum (ER), which help to promote TBEV genome replication and afford protection for newly synthesised TBEV RNA [54]. Upon host and viral proteases cleaving the polyprotein, the liberated NS1 protein forms hexameric complexes and assists in downregulating the complement mediated destruction of TBEV particles [55]. As in the case of Hepatitis C, the NS2-NS3 proteins have both protease and helicase activity, while NS4 and NS5 are involved in the downregulation of the cellular antiviral response. In addition, the NS5 protein encodes the RNA-dependent RNA polymerase (RdRp), which, alongside host transcription factors and promoters, helps to regulate TBEV replication [55]. Despite TBEV being able to infect multiple cell types, there is clear evidence for preferred neuronal tropism, with neuronal cells exhibiting 10,000-fold higher TBEV replication rates than epithelial cells [52]. Through the production of multiple replication complexes within infected cells, viral proteins are effectively concentrated, allowing for rapid virion production within ER vesicles [54]. Within the ER vesicle, maturation of the virion occurs through rearrangement of the preM and E proteins, and as they pass through the Golgi network, glycosylation of the E protein occurs, allowing for fusion with the cell membrane and virion release into the extracellular space [55].

## 3. Natural History of TBEV Infection

### 3.1. Initial Viraemic Phase

**Figure 3:**
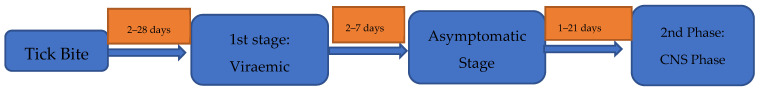
The typical clinical pattern and diagnostic methods employed in biphasic European TBEV [17,27,56,57,58].

As is the case with many tick-borne diseases, approximately a third of patients with TBE do not recall being bitten by a tick [56]. Moreover, around 75% of European TBE infections are asymptomatic [7]. The incubation period of TBE is typically 7–14 days (range 2–28 days); however, this can be significantly shorter in the setting of alimentary transmission at 3–4 days, making this an important exposure to consider when assessing TBEV risk [17,27,56,57,58] (Figure 3). The biphasic pattern of disease in European TBE is observed in only two thirds of symptomatic individuals [57]. Moreover, this pattern may occur in other viral and bacterial conditions, including Dengue, Zika [59], severe acute respiratory syndrome coronavirus 2 (SARS-CoV2) [60], leptospirosis [61], and pulmonary anthrax [62].

The first phase of European TBE is characterised by viraemia and typically lasts 2–7 days [17]. At this time, patients present with a flu-like illness, including fever, headache, myalgia, and, in a minority, diarrhoea and abdominal pain, with a spectrum of illness reported. Rarely, patients can present with severe inflammatory myositis. A similar clinical picture is seen in those with monophasic disease, although features such as ataxia, vomiting, and nausea are more pronounced in this setting (Table 1) [63,64,65,66,67,68,69,70]. Unlike in borrelia, rash is not a recognised manifestation of TBE and should prompt reconsideration of the diagnosis or the possibility of coinfection. During this first phase, thrombocytopenia, leukopenia, including lymphopenia, hyperbilirubinaemia, and hepatitis are commonly seen [63]. While the hepatitis is often mild, it can be moderately severe, with the serum alanine transaminase (ALT) and aspartate transaminases (AST) elevated up to 2–3 times the upper limit of normal and may take 3–4 weeks to normalise. Other described findings include hyponatraemia, hypokalaemia, and raised creatinine kinase [71]. After initial viraemia, an abortive course of TBE may occur, although the determinants of this disease phenotype remain ill-defined [72]. 

### 3.2. Neurological Phase—Characteristics and Presentation

After a disease-free interval of approximately 7 days (range 1–21 days), the second phase of disease, typified by CNS involvement, occurs. This second phase is reported in ~10% of individuals, although this is significantly higher in children (5–30%) and in those in whom alimentary transmission has occurred (~38.9%) [1,5,11,23,27]. When comparing TBE patients with or without second-stage disease, there appears to be no predictive first-stage symptoms or routinely collected laboratory markers. This means it is important that clinicians remain vigilant about the risks of second-stage disease and inform patients of its possible development [22]. 

Upon the onset of this second phase, hospitalisation often occurs due to worsening systemic symptoms, headache, photophobia, vertigo, tremor, and behavioural change [17,22]. During this second phase, the various cytopenias of the first phase resolve and often become only mildly elevated, alongside elevations in serum C-reactive protein (CRP) and Erythrocyte sedimentation rates (ESRs) in 80% and 90% of cases, respectively. The degree of biochemical derangement during this phase does not appear to influence the prognosis of TBE [73,74,75]. Overall, 37.3–50% of adult patients present with meningitis, while up to 51% present with meningoencephalitis, the most severe form of the disease. In a minority of patients (7–10%), myelitis is observed, often presenting as a flaccid paralysis of the upper limbs and neck, and it may be co-existent with meningoencephalitis [65,68]. A review of the Polish TBE registry between 1993 and 2008 demonstrated that meningoencephalitis is the predominant CNS presentation in patients >30 years old, while meningoencephalomyelitis prevalence increases and peaks at 50 years, before becoming less prevalent, suggesting that age is a risk factor for this disease phenotype [65]. Interestingly, a case of TBE presenting as meningoencephalomyelitis was reported in a 24-year-old female who had recently recovered from SARS-CoV2 infection, suggesting the possibility of immune enhancement of TBE CNS disease in this setting [76].

A spectrum of psychiatric symptoms are described during this phase, ranging from mild cognitive disorder in 31.9% to sleep disorder in 12.4% and overt psychosis in 2.1% [65]. A rare case of anti n-methyl-d-aspartate (NMDA) encephalitis has also been described in the setting of TBE, in a child who presented 1 month after initial resolution with apathy, mutism, and worsening hand tremor. While rare, this is an important consideration in patients with clinical worsening in the setting of TBE and adds to the multiple infectious causes of NMDA encephalitis, which include herpes simplex virus 1 (HSV1) [11].

Amongst other neurological presentations, chorea and cranial nerve palsies of the facial, ocular, and vestibular nerves are described, which may confuse TBE with Borrelia and autoimmune conditions [17,77]. In patients with meningitis and meningoencephalitis, peripheral and autonomic nervous system involvement may occur and is likely a significant contributor to both acute TBE mortality and long-term morbidity [78]. Overall, 12% of patients during the CNS stage require intensive care unit (ICU) admission, and 5% require assisted ventilation [1]. Of these presentations, meningoencephaloradiculitis predicts ICU admission and mechanical ventilation, with diabetes mellitus a possible risk factor for its development [79].

The ability to risk stratify and predict the severity of second-stage disease among TBE patients through suitable biomarkers is an area of great interest. In TBE patients, the presence of detectable serum matrix metalloproteinase 9 (MMP 9) has been observed to predict meningoencephalitis development [80]. Indeed, a single nucleotide polymorphism in the MMP9 gene, rs17576, was found to be significantly higher in patients with meningoencephalitis than those with meningitis (*p* = 0.042) [81]. Moreover, MMP 9, when detected within the CSF, was found to predict CSF TBEV IgG positivity, to correlate with elevated serum IL-6 concentrations, and to be associated with poorer outcomes, including death [82]. Importantly, serum MMP 9, while elevated in both adult and paediatric TBE cases versus controls, remained detectable until resolution of the disease [83]. Unsurprisingly, MMP 9 is felt to offer both biomarker and therapeutic opportunities [80]. 

Chemokines are cytokines that assist in leucocyte migration and thus inflammation and assist in coordinating the immune response. In TBE patients, serum concentrations of CXCL10 and CXCL13, which function as T and B-lymphocyte chemoattractants, respectively, are significantly increased versus controls and decrease on clinical recovery. However, these are not specific to TBE and are raised in enterovirus meningitis and neuroborreliosis [84]. Of cytokines, which have been shown to be predictive of TBE, CSF IL-6 in the acute phase of TBE is far higher than in neuroborreliosis or healthy controls and mirrors significant decreases in CSF IL-10, a known anti-inflammatory cytokine. Indeed, the CSF IL-6/IL-10 ratio has been found to be discriminatory in TBE but is an invasive test [80]. In a study by Palus M et al. (2021), the authors assessed the breadth of the acute immune response in 87 TBE patients versus 32 controls, including serum cytokines, chemokines, and unique growth factor concentrations [85]. In doing so, they identified that vascular endothelial growth factor (VEGF) and human growth factor (HGF) were significantly raised versus controls and likely reflected a host response to peripheral viraemia and tissue damage and could serve as a biomarker of TBE. Unfortunately, the authors did not have the clinical details available to correlate the levels of VEGF and HGF with underlying disease severity. Nonetheless, this result is of interest. Given that TBEV can replicate within neurons of the CNS, the measurement of recognised brain damage markers could be useful. Neurofilament light (NfL) protein and glial fibrillary acidic protein (GFAP) are markers of neuroaxonal and astrocyte damage, respectively. In a study of 115 TBE patients from Lithuania and Sweden, all patients had detectable CSF and serum NfL and GFAP, which were positively correlated with one another. Moreover, both markers were significantly elevated in patients with moderate/severe disease versus those with mild disease, which was independent of the patients age. This makes NfL and GFAP more attractive as biomarkers than the Tau protein, which does not appear to correlate with disease severity. However, while all blood draws in this study were performed within 2 weeks of hospital admission, the kinetics of these markers remain unknown and require further study [86]. In summary, it is likely that a combination of biomarkers will be needed to offer good discrimination in the diagnosis, prognosis, and outcome of TBE. Moreover, the need to ensure that putative biomarkers are accessible, cost-effective, and sensitive in the clinical setting will require considerable work. 

### 3.3. TBEV and Coinfection

Given the geographical overlap of tick-borne diseases, the possibility of coinfection is a real possibility. In endemic areas of Borrelia, 20% of cases have coexistent babeosis. These findings correlate with a large literature review analysing coinfection in the setting of TBE in Europe, where 41.3% (n = 273/655) of TBE patients had evidence of borrelia coinfection [20]. However, of borrelia diagnoses, 88% (n = 240) were made on the basis of seropositivity alone. As such, the inherent problems of differentiating between active/past borrelia infection and non-specific serological cross-reactivity are important confounders. Interestingly, two clinical patterns of likely TBE and Borrelia coinfection were described, including high fever and erythema migrans and the presence of early neurological symptoms [20]. 

In contrast, in a Polish review of 110 patients with TBE, coinfection rates, as determined by serum/cerebrospinal fluid (CSF) polymerase chain reaction (PCR) with Borrelia, human granulocytic anaplasmosis (HGA), and Babesia, were lower at 27%, 10.9%, and 0.9%, respectively. Triple infection (TBEV, Borrelia, and anaplasma) was detected in 2.7% of patients [21]. When assessing the initial symptoms of patient TBE versus in those with or without coexistent infections, no overall differences in clinical presentation were described, which concurs with the literature. Biochemically, however, ESR was found to be significantly raised in mixed infections (45 mm/h vs. 34 mm/h, *p* = 0.028), while serum ALT was lower (25 U/L vs. 34 U/L, *p* = 0.006) [21]. While coinfection with Borrelia and Babesia is linked with worse clinical outcomes, this has not been observed in Borrelia and TBE coinfection. However, once more patients with borrelia coinfection were more likely to present with neuroborreliosis, suggesting an alteration to disease phenotype through an already damaged blood–brain barrier (BBB) [87,88,89]. As such, suspicion of coinfection should be raised where there is a lack of clinical response to treatment, e.g., doxycycline in Borrelia infection or mixed symptomatology, in an area where other tick-borne diseases including TBE are present. In such cases, investigations for coinfection should preferentially utilise molecular testing in place of serology. 

While the ability to detect tick-borne disease coinfection based on biochemical markers is highly attractive, they may also be able to assist in differentiating primary tick-borne diseases while awaiting confirmatory diagnostics. In this regard, one review compared serum CRP and procalcitonin (PCT) in patients with first-stage TBE and HGA. It was noted that, in TBE, (n = 17) < 20% of patients had a raised CRP (average 5 mg/L; range 3–16 mg/L, normal < 5 mg/L), and all HGA patients (n = 11) had an elevated CRP (average 121 mg/L; range 15–314 mg/L) (*p* < 0.001). Serum PCT exhibited a similar pattern at 2.5 µg/L vs. 0.121 µg/L in HGA and TBE, respectively (normal < 0.5 µg/L) (*p* < 0.001). This yielded a positive and negative predictive value of PCT and CRP of 100%/79% and 94%/100%, respectively [90]. In contrast, in Borrelia infection, the majority of patients with acute localised/disseminated borrelia have a normal PCT, while the CRP is often mildly raised pre-treatment versus post treatment and in healthy controls [91,92]. As such, further study of the patterns of acute phase reactants amongst tick-borne diseases is an important future research area that can be used to assist in assessing patients presenting with undifferentiated fever after a tick bite.

### 3.4. Diagnosing TBEV

The diagnosis of TBE is dependent on the stage of the disease in which the patient presents. In the first phase of the disease, characterised by viraemia, serum, and very rarely CSF TBEV reverse transcriptase-polymerase chain reaction (RT-PCR) is positive, in the absence of serology [1,93]. In the setting of immunosuppression, peripheral viraemia may persist for up to 74 days post-infection [93]. As demonstrated in other orthoflaviviruses, urine can be a potential diagnostic sample. Indeed, while urine is seldom tested, there is evidence that urine TBEV PCR can be positive in both immunocompetent and immunocompromised individuals, even during the second phase of disease. Given the obvious advantages of urine versus blood collection, this diagnostic method requires further study [17,94,95]. Importantly, there is no appreciable relationship between serum TBEV viral load and patient age, sex, biochemical parameters, disease severity, CNS presentation, or outcome [3,93]. 

With the development of the second phase of disease, the viraemia disappears with serum, and on occasion, CSF TBEV IgM and IgG now detectable. While TBEV IgM typically persists for 6 weeks and up to 10 months, TBEV IgG peaks at 3–7 weeks post-infection and persists for years [17,80]. Detection of TBEV IgG and IgM may be performed by utilising commercial enzyme-linked immunosorbent assay (ELISA) tests, which are favoured when compared to haemagglutination tests due to lower associated risks of flaviviral cross-reactivity. In the 5% of patients who do not have detectable serum TBEV IgG at the time of neurological onset, they have a twenty-times higher risk of meningoencephalomyelitis development and subsequent post-encephalitic syndrome [80]. As such, predictive factors for severe neurological disease include low neutralising serum TBEV IgG antibody titres alongside low early CSF IgM response [80,96]. A review of notified TBE cases from the Public Health Agency in Sweden analysed the accuracy of TBE serology and RT-PCR diagnostics. Of the 111 reported TBE patients, 98.2% (n = 109) had positive serum IgM, while CSF IgM was positive in a third (29/88) versus controls, in which 99.3% (n-139/140) were negative [97]. 

To assist in diagnosing CNS disease, CSF analysis should be undertaken via a lumbar puncture if no contraindications exist. As is typical in viral CNS disease, a normal serum:CSF glucose ratio is observed with a normal CSF lactate (<2 mmol/L); although, this may be mildly raised in the setting of meningoencephalomyelitis [57,68]. Two thirds of patients with TBE have a lymphocyte pleocytosis of ≤100 cells; however, normal cell counts in the setting of TBE encephalitis have been described, as well as markedly raised cell counts of up to 1200 cells [1,5,17,75]. As such, a normal CSF white cell count, while reassuring, cannot rule out TBE. CSF protein is often elevated; in one case series of 81 TBE patients, 82.7% had raised CSF protein levels (range 0.53–0.91 g/L), with the CSF/serum albumin level recorded at 10.56 × 10^−3^ (normal < 7.5), highlighting BBB dysfunction [73]. 

## 4. Immunology and Pathophysiology of TBEV

### 4.1. Peripheral Immune Responses to TBEV

Upon attaching to a host, an infected tick will transmit TBEV in its saliva almost immediately, making early tick removal often ineffective in preventing TBEV transmission [1,5,98]. Tick saliva itself has several properties that facilitate early infection including antihaemostatic, vasodilatatory, and local immunomodulatory activity [98]. Part of this immunomodulation includes decreased natural killer (NK) cell activity, attenuation of the antigen presentation activity and cytokine production of dendritic cells (DC), Langerhans cells and macrophages, reduced recruitment of macrophages to areas of inflammation, and the promotion of ineffective Th2 cellular responses [98,99]. 

During initial skin infection, TBEV will replicate within the subcutaneous tissue, primarily within Langerhans cells and keratinocytes, but it may also do so in resident mast cells, fibroblasts, and monocytes. This tropism allows for local dissemination of TBEV and transport to local lymphoid tissues, where systemic spread and involvement of cells of the adaptive immune system may occur [100,101,102,103]. Alongside this cellular spread, tick saliva enhances the expression of pro-oxidative proteins, such as glutathione and 4-hydroxy-nonenal (4-HNE), which are known to cause protein adducts and are significantly increased in the plasma of TBE patients versus controls. This pattern of oxidative stress and downregulation of protective antioxidant systems leads to cellular damage and perpetuation of non-protective inflammatory responses while enhancing tumour necrosis factor alpha (TNFα) production [6]. Upon cellular infection, TBEV is identified by its pathogen-associated molecular patterns (PAMPs) by cell pathogen recognition receptors (PRRs), which include RIG-like receptors and Toll-like receptors (TLRs). TLR7 and TLR9 recognise ssRNA and cytidine phosphate guanosine (CpG) sequences, respectively, with CpG sequences highly expressed within the TBEV genome. Following TLR activation, NF-kB production and proinflammatory cytokine production occur, culminating in interferon-stimulated gene expression. The production of interferon stimulates >300 proteins, which assists in priming cellular and local antiviral immunity through several mechanisms, including autocrine/paracrine signaling, enhanced antigen presentation, interruption of multiple aspects of the viral life cycle, and activation of neighbouring macrophages, NK cells, and cells of the adaptive immune system. The importance of interferon in TBEV infection is demonstrated by the 1000-fold reduction in TBEV infection in interferon-pre-treated cells [98,101,103,104]. As one would expect, deficiencies in this initial response may be deleterious to the host, with evidence that differences in TLR expression, namely polymorphisms of DC and epithelial cell expressions of TLR3, can predispose to both symptomatic and severe forms of TBE [105].

TBEV, however, has methods to circumvent host antiviral responses. The TBEV NS5 protein attenuates this process significantly by decreasing expression of the IFN receptor subunit, IFNAR1, and downregulating JAK-STAT signaling pathways, allowing for permissive TBEV replication [49]. Because of this, prolific immune cell activation can occur, leading to grossly elevated serum pro-inflammatory cytokines (e.g., IL-6, IL-8, IL-12, IL-17A) and chemokines (e.g., IL-8, CCL2, CCL5). This results in inflammation and damage to local tissues, including the BBB, allowing for subsequent immune cell infiltration of the CNS and the development of neurological disease [6,104]. The chemokine receptor 5 (CCR5) protein normally functions as a ligand for CCL3 and CCL5 chemokines, aiding in lymphocyte migration to the CNS, and is a key co-receptor for human immunodeficiency virus immune cell entry [106]. While homozygote/heterozygote CCR5Δ32 deletion was thought to predict symptomatic and severe forms of TBE by means of a lack of robust lymphocyte activity, this has since been disproved on further study [105,107,108]. Therefore, it is important to consider, when studying biomarkers, the underlying genetic differences in the population cohorts utilised.

NK cells are an important component of the innate immune response and early antiviral response functioning in an antigen-independent manner. Reduced NK cell activity is predictive of worse outcomes in Dengue infection [3,52]. NK cell activation during TBEV infection, as measured by serum Ki67 expression, is highest within the first week of symptoms, where viraemia is present. However, mouse models suggest that NK cell activation is transient and is followed by NK cell suppression [3]. In TBE patients, activated NK cells express less perforin and granzyme, making them less cytotoxic and, as such, unable to abort or control the infection. NK cells may be detected in the CSF during the second phase of the disease, although their role in CNS disease is unclear [52]. Therefore, the development of the adaptive immune system is likely key in controlling TBEV infection due to their antigen specificity. However, as TBEV attenuates DC maturation, migration, and cytokine production (e.g., IL-15, IL-12), the abilities to present antigen, costimulate, and propagate Th1 type responses are greatly reduced. [98,109]. In in vitro experiments, individuals with breakthrough infections after vaccination have also shown to have weaker DC and macrophage cytokine responses upon TBEV challenge [110]. 

Of T-cell responses, CD8+ T cells are the most important and demonstrate cytotoxicity upon recognising antigens bound to surface human leucocyte antigen (HLA) class 1 molecules. Upon activation, CD8+ T cells will proliferate and be sustained as a small pool of circulating CD8+ T-cell memory cells protecting the host from reinfection. On assessing TBEV CD8+ T cell trends in 20 TBE patients, it was found that they were undetectable upon hospitalization and peaked by day 21, where maximum serum IFNγ and TNFα expression was noted [111]. Upon further analysis of CD8+ T cell responses, distinct TBEV epitopes and phenotypes have been recognised [111], typically active against TBEV NSPs (NSP1, NSP5), indeed temporal changes in the CD8+ T cell phenotype during TBE infection has been observed from CD45RA-CCR7-CD27+CD57- at day 7 infection to CD45RA-CCR7-CD27+CD57+ at day 21 infection [112]. Therefore, further understanding of TBEV CD8+ epitopes may be useful, allowing future TBE vaccine design to facilitate robust adaptive immune responses and prevent breakthrough infection [113]. Out of immune responses, it has been demonstrated that robust innate and Th1 immune responses (e.g., IL-1β, TNFα) are positively and significantly associated with disease severity and outcome, while Th17 (e.g., IL-17, IL-22) and B cell responses are less implicated [73]. Like in other orthoflaviviruses, e.g., Dengue, antibody-dependent enhancement (ADE) may be implicated in explaining the spectrum of disease in TBE patients, with evidence of Fcγ receptor independent mechanisms of ADE uniquely described in this population [55]. 

### 4.2. CNS Entry of TBEV

A commonality amongst orthoflaviviruses is their neuroinvasiveness and neurovirulence [100]. While many flaviviral infections are inapparent, CNS disease is more likely to appear in patients who are elderly and comorbid (e.g., chronic kidney disease) and in individuals who are immunosuppressed [114]. TBEV is highly adapted to replicating efficiently in neuronal cells, where it can reduce their viability and transmit trans-synaptically to infect nearby neurons [52,100]. TBEV may also infect and replicate within astrocytes and glial cells, which support neuronal metabolic and immunological functioning [115]. In in vitro models, oligodendrocyte infection has also been observed; however, it is unclear if this occurs in vivo and, if so, how this contributes to TBE CNS pathology [115]. 

**Figure 4:**
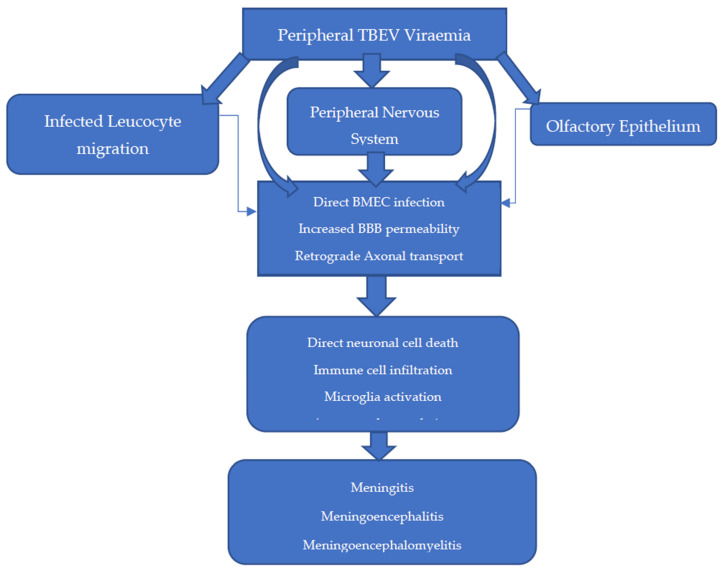
Proposed methods of entry into the central nervous system (CNS) [3,98,100,105,116].

The entry of TBEV into the CNS is theorised to occur in several ways, with infection and replication in brain microvascular endothelial cells (BMECs) and retrograde axonal transport likely the most significant [3,98,100,105,116] (Figure 4). The BBB is a semipermeable anatomical structure, composed of non-fenestrated BMECs joined in an uninterrupted fashion by tight junctions with supportive pericytes and astrocytes. It functions primarily to maintain cerebral homeostasis and maintain the immune privileged state of the CNS. Recent in vitro models demonstrate that transcytosis of WNV and JEV across BMECs is possible, suggesting that altered BBB permeability is not always a pre-requisite to CNS infection [98,110,117,118]. Indeed, in a BALB/c and C57BL/6 mouse model of TBE, breakdown of the BBB was observed, but this was a late feature of disease after TBEV was already detectable in the brain and was not CD8+T-cell-dependent [119].

While comprehensive, the BBB is deficient at several anatomical sites, including the choroid plexus, circumventricular organs, and post-capillary venules, and thus, these are areas where TBEV infiltration can occur during viraemia [3]. This likely explains the predilection for lesions to develop in and around these locations, as assessed by brain magnetic resonance imaging (MRI) [120]. 

In other areas of the BBB, TBEV entry into the brain can occur because of enhanced permeability by several methods. Firstly, the NS1 protein can directly influence BBB permeability through the activation of sialidases and heparinases. Secondly, astrocyte infection and macrophage activation can lead to oxidative stress, resulting in reduced tight junction expression through metalloproteinase activation (e.g., MMP 9) and disintegrin upregulation. Thirdly, the proinflammatory cytokine environment (e.g., IL-6, IFNα, IL-8) can enhance TBEV-infected immune cell diapedesis into the CNS [80,117,118]. 

Retrograde transmission from nerves of the peripheral/autonomic nervous systems has been observed in the setting of WNV in rhesus monkeys and in in vitro models of TBEV [78,118]. This route may explain the method by which alimentary TBEV transmission leads to CNS disease and the method by which delayed TBE CNS disease presentation occurs in infected mice upon transection of the sciatic nerve [100]. The olfactory bulb is a neuronal structure vulnerable to infection due to its exposed nerve terminals within the olfactory epithelium [121,122]. In a historic mouse model of SLE, olfactory transmission was reported after intranasal exposure. However, upon closer inspection, this coincided with splenic involvement, suggesting more likely that haematogenous spread was involved [123]. While evidence of the olfactory transmission route is conflicting, there has been four reported cases of aerosol transmission of TBEV in a laboratory setting, which in one case led to fatal meningoencephalitis [124]. While likely a low transmission risk to the public, this finding may therefore have implications in handling and standard operating procedures in the laboratory of European TBEV, which is currently a category 3 pathogen [125]. 

### 4.3. CNS Pathophysiology

Upon entry into the CNS, TBEV propagates and elicits downstream changes, which lead to clinical disease. TBEV may directly kill neurons, with the necrosis of neurons observed in vitro [126]. Furthermore, there is evidence showing that orthoflaviviruses can affect neuronal stem cell populations and lead to cell cycle arrest and thus could explain persistent neurology after acute disease [118]. The major mechanism of CNS dysfunction is likely from immune cell infiltration into the CNS, which provokes neuronal cell death and inflammation. These processes further effect microglia and astrocyte populations, with quantitative deficits in microglia linked with mortality in viral encephalitis [4,127]. 

Upon histopathological analysis of the CNS from fatal human TBE cases, neuronal loss, reactive gliosis, neuronophagia, and dense immune cell infiltration were observed in the thalamus, cerebellum, caudate nucleus, and brainstem [17,128]. Of the immune cell populations noted, macrophages, CD4+ cells, and CD8+ T cells are the most common, with CD8+ T cells considered the most pathogenic given the high levels of β2-microglobulin, a marker of CD8+ T cell activity, found upon histopathological examination [98,119]. In TBE mouse models, CD8+ T cell knockout and severe combined immunodeficiency (SCID) mice lived significantly longer than controls, with a similar observation observed in the setting of other orthoflaviviral infections. This highlights the significance of CD8+ T cells in TBE pathogenesis [98].

Imaging of the brain in TBE often shows nonspecific changes. Computed tomography (CT), while readily available in most well-funded healthcare systems often shows no abnormality. Described findings on CT include non-enhancing hypodense lesions within the thalamus and putamen [129]. While PET-CT is rarely utilised in encephalitis workup, unusually in TBE, focal and, in rare instances, global decreases in fluorodeoxyglucose uptake have been described [130]. MRI is the preferred radiological study for the assessment of encephalitis, given its superior ability to demonstrate the degree of cerebral involvement and provide aetiological clues [131]. In a case series of 12 TBE patients with meningitis or meningoencephalomyelitis, MRI of the brain performed within the first 3–5 days from diagnosis demonstrated non-enhancing subcortical and periventricular lesions, with four cases of cortical atrophy noted. Overall, only 18% of patients demonstrate MRI changes [132]. Importantly, a range of contrast-enhancing lesions has also been described within the meninges, cerebellum, and anterior horns of the spinal cord. While these findings are present in other causes of encephalitis, diffuse signal hyperintensities of the crura cerebri seems relatively specific for TBE. Furthermore, MRI studies have demonstrated alterations to cerebral perfusion, with evidence of significant increases in cerebral blood flow and blood volume noted within the thalamus. These radiological changes in the majority of cases appear to be transient, with the majority resolving within 16–34 weeks, even in the presence of ongoing symptoms [132,133]. However, in those with severe TBE disease (encephalitis/encephalomyelitis), imaging changes may persist for a significant time, with CT of the head at 10 years post-diagnosis demonstrating accelerated localised cerebral atrophy in the setting of persistent electroencephalographic (EEG) changes [132,134,135].

### 4.4. CNS Antiviral Responses

TBEV elicits different antiviral responses within cells of the CNS. In an in vitro model, it was found that astrocytes upregulate immune gene expression earlier, at greater levels, and more durably than in human progenitor neural cells, leading to enhanced interferon λ1/2 production [4]. This may underpin the increased susceptibility of neurons to TBEV infection and suggests that, while astrocytes afford some protection to surrounding neurons, they cannot avert neuronal infection and instead can contribute to neurotoxicity via cytokine release [116]. This disparity in CNS cellular responses is further evidenced by differences in micro ribonucleic acid (miRNA) expression. Indeed, infected neurons have been found to upregulate hsa-miRNA-1298, which is known to downregulate proteins that can reduce TBEV replication [4]. As miRNAs are potent regulators of gene expression, they may represent a pharmacological target for boosting host immunity. In one study, multiple TBEV virus subtypes (e.g., Langat virus) were modified to include miRNA124a and miRNA9 transcripts at their 3′ NCR before being introduced into SCID mice and healthy controls. While all immunocompetent mice demonstrated no observable neurological disease, SCID mice demonstrated significant reductions in viraemia and delays in the presentation and progression of paralysis [136]. Importantly, in those SCID mice who became symptomatic and died, evidence of large genomic deletions within the 3′NCR were noted, which were likely because of unrestricted peripheral TBEV proliferation, low-fidelity RNA polymerase, and thus genomic instability. As such, this may be a novel approach for vaccine design in the immunocompetent host, with animal studies highlighting the promise of this approach and its feasibility for future human trials [137].

Within the CSF, intrathecal antibody production occurs in TBE as it does in other orthoflaviviruses, and in WNV, CSF IgM may be present for up to 199 days [138]. While the presence of CSF antibody in TBE could be a byproduct of increased BBB permeability, evidence of intrathecal production includes increased CSF λ and κ light chains when compared to the serum in patients pre-treatment and after recovery [80,139]. This production is linked with increases in B-cell chemokines of the CSF, including CXCL12, CXCL13, and IL-5, which assist in the differentiation of B-cells into plasma cells [140]. In conjunction with this, intrathecal complement synthesis also occurs in the setting of acute TBE, with statistically significant increases in C1q, C3a, C3b, and C5a observed. While CSF complement levels were highest within the first week from fever onset in 20 TBE patients, they remained elevated for 3 months post-disease onset, but they were not linked with the development of neurological sequelae [141]. When evaluating CNS cytokine responses in both human and animal models, the earliest upregulations include granulocyte-colony stimulating factor (G-CSF) and CXCL10, which is important in recruiting CD8+ T cells. Unsurprisingly, CXCL10 expression was found to correlate with CNS disease phenotype and outcome by virtue of its ability to upregulate IL-1β,4,6,8,10, and 17 and, in doing so, sustain immune cell infiltration and inflammation to try and reduce TBEV replication [142].

## 5. Outcomes and Management

### 5.1. Long-Term Neurological Effects and Impact on Quality of Life 

The outcome of TBE infection is variable, but it seems heavily dependent on the viral strain, disease phenotype, and host immune response. Of presentations, meningoencephalitis and meningoencephalomyelitis predict the worst clinical outcomes. Of host characteristics, male gender and older age at the time of disease onset are linked with long-term morbidity [17,79]. Overall, while the mortality rate of European TBE is low, the associated morbidity is significant [21,79]. In a recent review of 523 TBE paediatric and adult cases, full recovery was noted in 94.9% and 63.8% of children and adults, respectively [143]. In 2016, the Swedish institute for disease control and prevention performed a retrospective study analysing long-term outcomes in 96 TBE patients (median age 59 years), 2–15 years post mild to severe CNS disease. Mild disease (35%) was defined as meningeal signs with normal EEG, while moderate (56%) described moderate encephalitis with slightly altered consciousness, and severe (7.3%) disease included severe encephalitis with multifocal symptoms. When comparing outcomes, increasing disease severity was associated with significant increases in balance disturbance and face/name and short-term memory impairment. Moreover, when compared to healthy controls, as assessed by the Encephalitis Support Group Questionnaire 2000 (ESGQ), TBE survivors reported significant differences in long-term memory (3.65 vs. 3.68, *p* = 0.028), learning difficulties (3.50 vs. 3.90, *p* = 0.027), motivation (3.49 vs. 3.76, *p* = 0.033), fatigue (3.14 vs. 3.60, *p* = 0.019), and fine motor skills (3.82 vs. 3.97, *p* = 0.040) [144]. This highlights the profound level of disability that persists in TBE survivors and thus requires long-term follow up in order to mitigate. A rare case of development of anti-GAD-mediated stiff person syndrome has been reported post-TBE meningoencephalitis, which is also of concern [145]. 

It is of no surprise, given the degree of disability from TBE, that there are significant societal and direct/indirect health-related costs. In a review of Swedish cases between 1998 and 2014, adult survivors required, on average, 42, 53, and 49 days of sick leave from work within the first 1, 3, and 5 years post-TBE diagnosis. This often led to early retirement, with associated healthcare and sick leave costs at 1, 3, and 5 years of EUR 20,504, EUR 24,126, and EUR 21,834, respectively, per TBE case [146]. This makes TBE on par with HSV-1 encephalitis morbidity, in which 60% suffer epilepsy and 22% require disability pension [147,148]. As such, preventing TBE and producing effective disease-modifying treatments are key to addressing the anticipated increases in TBE cases and improving long-term outcomes. 

### 5.2. Current Treatment Options

Current management of TBE is supportive and involves the use of antipyretics, analgesia, intravenous fluid, and anticonvulsive agents [17]. Alongside this the measurement and control of intracranial pressure (ICP) is required to prevent disability and death and includes head-up positioning, hyperventilation, hypertonic saline, and intravenous mannitol [149]. Mannitol is an osmotic diuretic that reduces sodium and water reabsorption in the proximal convoluted tubule and loop of Henle, thereby reducing cerebral oedema [149]. However, mannitol use is not without controversy, given the lack of robust evidence that it improves neurological outcomes and its inherent risks, i.e., severe hypernatraemia and kidney failure [150]. In a study looking at the biochemical effects of a single dose of 0.25 g/kg 15% mannitol in TBE patients, high rates of hypokalaemia and hypernatremia were observed in older men and persisted for 7 days post-use. Moreover, in those with meningoencephalitis and meningoencephalomyelitis, the development of syndrome of inappropriate anti-diuretic hormone (SIADH) was observed [151]. While the authors suggested that mannitol could be utilised up to four times a day, they did not assess clinical status as a primary outcome, making this suggestion contentious. The European Academy of Neurology (EAN) suggests that, if utilised, mannitol should be administered as bolus therapy for a maximum of 1–2 days rather than continuously [5]. Indeed, the use of osmotherapy agents in TBE has been identified as a key area for further research in an attempt to improve TBE neurological outcomes [152].

The use of dexamethasone and other glucocorticoids is currently not advocated in TBE, given the lack of efficacy and the observation that their use prolongs hospitalisation [6]. In a Polish review of 687 TBE patients presenting during 1993–2008, 407 patients received dexamethasone as part of their treatment [65]. Overall, patients received between 6 and 32 mg of dexamethasone for a median of 9 days (range 1–64 days), with patients with meningoencephalomyelitis more likely to receive higher dosages. While, anecdotally, this was linked with clinical improvements, including the resolution of fever, it did not lead to quicker resolution of CSF/serum biochemical abnormalities and often necessitated repeat lumbar puncture examinations due to prolonged hospitalisation [65]. As such, steroids should not be empirically given in the setting of TBE.

Tetracyclines are a class of antibiotics that inhibit bacterial protein synthesis and demonstrate potent anti-inflammatory action. Doxycycline is currently recommended in the setting of several tick-borne diseases as post-exposure prophylaxis and treatment, where it reduces attendant mortality and morbidity [153]. However, the use of tetracycline in TBE remains questionable, with few studies ultimately informing its use. In one small Russian study, 29 patients were randomised to conservative treatment, which included intravenous immunoglobulin (IVIg) administration or conservative treatment alongside tetracycline use at 250 mg QDS or 25 mg/kg in adults and children, respectively [154]. In those who received tetracycline, a quicker resolution of disease and shorter hospital stays were seen, although this did not reach significance. While tetracycline use may therefore be used in the setting of TBE and Borrelia coinfection, there are similar rates of incomplete recovery with its use versus controls [155]. 

While the use of IVIg to treat and attenuate TBE disease has been limited in Europe due to the concerns of ADE, in Russia, it remains standard practice. In this setting, IVIg is given within the first four days post-tick bite as a method of post-exposure prophylaxis, with encouraging safety and efficacy data from animal studies [156,157]. The use of IVIg, however, may cause several important side effects, including anaphylaxis and thromboembolic events, although slow intravenous administration and preliminary screening for IgA deficiency may significantly reduce these risks. Within WNV and JE, IVIg has exhibited disease-modifying properties when administered over a five-day period and is an area of ongoing study [158]. However, given that IVIg is a non-specific pooled preparation from thousands of healthy donors, it is important that donors who have previously been vaccinated or infected be used to ensure that high levels of neutralising anti-TBEV IgG antibodies are present [159]. The use of human neutralising monoclonal antibodies circumvents this issue, with two preparations, T025 and T028, that target domain III of the E protein, able to prevent TBE disease in mice when used within 4 days of experimental infection while also demonstrating activity against other orthoflavivirus infection [160]. While TBEV escape has been documented with the individual use of T025 and T028, these escape mutants are far less pathogenic, which is encouraging. Importantly, such escape mutations are prevented when a combination of T025 and T028 monoclonal antibodies is used [161]. As such, monoclonal antibody therapy represents an exciting and viable management option in TBE and is an ideal candidate for human trials. 

### 5.3. Antiviral Therapeutic Landscape in TBE

At this time, there are no antiviral treatments recommended for use in TBE, with novel drug discovery complicated by the paucity of established drug targets and the needs to be cost-effective and achieve adequate CSF penetration. In general, the majority of antivirals work to inhibit viral polymerase and thus genome replication. Previous work by Eyer et al. (2015) highlighted several promising nucleoside agents, including 7-deaza-2′-C-methyladenosine (CMA), which had in vitro activity against TBEV [162]. Importantly, they also found that intraperitoneal 7-deaza-2′-CMA administration showed efficacy in BALB/c mice infected with a lethal dose of the European TBEV strain, Hypr. Here, it demonstrated an impressive survival benefit of 35%, 50%, and 60%, at doses of 5 and 15 mg/kg once daily and 25 mg/kg twice daily, respectively, versus controls. Moreover, while an S603T escape mutation affecting the NS5 protein was found to markedly decrease 7-deaza-2′-CMA efficacy, increasing the 50% effective concentration (EC_50_) by 50-fold versus wild type virus, this mutation was associated with a drastically attenuated virus that was significantly less neurovirulent and associated with decreased mortality [163]. While 7-deaza-2′-CMA has been investigated in other orthoflavivirus infections, such as Zika virus, no human trials yet exist in TBEV, which should be encouraged [164]. 

When testing antiviral compounds, it is important that the in vitro model reflects the anatomy of the infection in vivo. Given that different cell lines may provide different results, a novel rat organotypic cerebellum slice (OCS) model, serving as a more complex in vitro model, was developed, utilising several potent nucleoside analogue compounds, including 7-deaza-2′-CMA [165]. Of these, 2′-CMA and 7-deaza-2′-CMA at a concentration of 50 µM demonstrated the greatest activity, reducing TBEV viral replication by 75–80 fold by quantitative PCR and by 10^3^ by plaque reduction assay. Importantly, both agents demonstrated no observable cytotoxicity up to 500 µM [165]. While this suggests that the OCS model may represent a faithful in vitro model, it is important to recognise that they cannot replace suitable in vivo models, where pharmacokinetics and pharmacodynamics are important considerations. Other antiviral approaches include fluorinated nucleoside analogues, which, due to their strong negative electrical charge, induce conformational change within the pentose sugar ring, making them unusable by viral DNA/RNA polymerase and resistant to intracellular degradation [166]. Of studied agents, 3′-deoxy-3′-fluoroadenosine in particular has shown efficacy against multiple orthoflaviviruses and TBEV strains. At low concentrations (1.6 µM), it can lead to sustained inhibition of viral replication and cytopathic effects for at least 72 h in porcine kidney stable (PS) cells. Importantly, this effect was most pronounced when PS cells were pre-treated 24 h prior to TBEV infection, raising the possibility of its use as a post-exposure prophylaxis in the clinical setting. Within a BALB/c mouse model infected with TBEV, intraperitoneal treatment with 25 mg/kg of 3′-deoxy-3′-fluoroadenosine for 6 days increased the mean survival of mice by 10.5 ± 1.9 days, but it did not reduce mortality, making this an important area of future research [166]. 

Repurposing existing antimicrobial compounds to treat TBE is advantageous given its inherent cost saving and pre-existing safety data. Novobiocin, for example, an anti-staphylococcal antibiotic, can inhibit Zika protease activity and improve survival in a mouse model; however, its use is precluded by its poor safety profile in humans. Other agents include the anticancer drug Sunitinib, which has shown some efficacy in Dengue animal models, but is limited in TBE due to its poor CNS penetration. In contrast, Niclosamide, an anthelminthic agent, can block E protein fusion with the host cell membrane and so could have broad potent anti-orthoflaviviral activity [167]. Specifically in TBEV, in vitro data exist for the use of Teicoplanin and Ivermectin, which inhibit the TBEV helicase and the E protein, respectively, at the possible expense of selecting for resistance in their traditional uses as an antibiotic and anti-helminth agent [156]. Galidesivir is an adenosine analogue originally used in Hepatitis C and is being trialed in Ebola and Zika, given its broad spectrum of RNA viral inhibition and lack of cytotoxicity [156]. Importantly, in the setting of a TBE BALB/c mouse model, Galidesivir not only proved to have disease-modifying properties, but also encouragingly, with the emergence of resistance, a loss of TBEV viral fitness and neurovirulence was found. This property, therefore, is highly desirable, and as such, Galidesivir is currently the subject of phase 1 trials in TBE [167,168,169].

Guanosine quadruplexes (G4) are common to several viral families, including herpesviruses and TBEV, and they are implicated in the initiation of genome replication and sustaining viral latency. Within TBEV, G4 complex targeting was found to reduce viral replication 10^3^-fold in PS cells, although issues with poor cellular uptake and cytotoxicity were observed [170]. While promising, these results, as with other trialed antivirals discussed, suggest that they need to be developed alongside suitable drug carriers, e.g., liposomes, to assist in CNS delivery and cellular uptake. 

## 6. Vaccination and Prevention of TBE

Given the spread of TBEV to UK shores, the evaluation of suitable public health measures is required. Within Austria, vaccination has been a cornerstone of reducing symptomatic cases of TBE by providing pre-existing immunity. Indeed, TBE vaccination increased in Austria from 6% in 1980 to 82–85% thirty years later, with cases declining by approximately 85% as a consequence [171,172].

Currently, there are two inactivated European vaccines utilised, namely Encepur (German) and FSME-Immun (Austrian). Both vaccines are well tolerated and licensed within children and adults. The vaccination schedule for both vaccines includes three primary doses within the first year, with booster doses at 3 years, and additional doses advised in those <5 years and >60 years to protect against waning immunity. Overall, seroconversion rates range from 86 to 100%; however, this is dependent on age, the presence of comorbidities, and the underlying trial design [173,174]. Breakthrough infection in vaccinated individuals is well described and is thought to represent 1.7–5% of all TBE cases and most commonly occurs in patients >50 years old or with an incomplete vaccination record [174,175]. Importantly, in breakthrough infection, there is no evidence of attenuated disease or more severe disease presentations to suggest ADE. However, this may be limited by the rarity of vaccine breakthrough cases and their inclusion in prospective and retrospective analysis [175]. To improve vaccine immunogenicity and provide a longer duration of protection, different means of targeting both the E protein and M protein are required. For this reason, improving T-cell responses would be useful, given their role in humoral responses, with recombinant vaccinia virus expressing the NS1 gene providing safe, effective, and durable responses against lethal TBEV challenge in a mouse model [176]. As highlighted previously, the use of miRNA-integrated vaccines may circumvent existing issues with current vaccine design, with single doses demonstrating excellent efficacy in mice [137]. Given that the Ixodes tick is intimately associated with sheep, cattle, deer, and a multitude of other hosts, the need to test and vaccinate animals is an important consideration but technically challenging if not impossible. 

A 2018–2019 UK seroprevalence study tested five culled deer species for TBEV or Louping ill serology by ELISA [43]. In total, 1323 deer were analysed with ELISA positivity for TBEV ranging from 0% in Somerset to 51.43% in Norfolk, England. Importantly, deer populations from nearly all studied areas of England as well as Scotland tested positive for TBEV serology [43]. This highlights not only that TBEV is indeed present within large areas of the UK but moreover that there are likely endemic hotspots that require further delineation to comprehensively evaluate the risks to the human population. At present, four UK cases of TBE, spanning from July 2019 to October 2022 and located in the New Forest, Hampshire, Loch Earn Scotland and North Yorkshire Moors, have been reported [16]. Therefore, the need to travel outside the UK is no longer mandatory for TBE testing and so should be considered on a case-by-case basis, particularly in the setting of aseptic meningitis or meningitis/encephalitis of unknown aetiology. 

Given this, the need for a TBE vaccination within the UK is uncertain, given the limited understanding of its epidemiology and distribution to make sound economic evaluations. To inform this decision, we require robust surveillance program data, cost effectiveness studies utilising quality adjusted life years (QALYs), incremental cost-effectiveness ratios (ICERs), ecological studies, and seroprevalence studies in high-risk groups, including farmers and forest workers. Increasing awareness of TBE amongst the public and healthcare professionals is therefore both important and required, given that Lyme disease has to date been considered the only significant vector-borne disease in the UK [177]. Good advice for the public and high-risk workers includes avoidance of the consumption of unpasteurised milk, to wear long-sleeved light colours, thus making ticks more noticeable, and avoiding walking through long grass and vegetation, particularly in the summer months. 

## 7. Conclusions

TBEV is an orthoflavivirus primarily spread by the Ixodes tick and may cause severe neurological sequelae, which may persist for years after clinical recovery. It is clear that host characteristics and immune responses are key in shaping and defining the disease phenotype with several potential biomarkers, including IL-6, CXCL9, MMP 9, and NfL, implicated. Like other arboviruses, the effects of climate change have led to altered epidemiology and distribution of TBE, with new areas of endemicity reported. Our ability to predict new areas of endemicity remains in its infancy, and improved understanding of the dynamics of TBEV transmission are required. While immunisation has been utilised in Europe for decades, breakthrough TBE in vaccinated individuals is reported, with several host and immune characteristics potentiating this. Given the detection of TBEV in the UK, awareness of this condition amongst healthcare professionals and the wider public is instrumental in its control. Through concerted efforts, including improvements in vaccine design and vaccination uptake, personal protection measures and environment modification may limit anticipated cases. Future work is required to help prognosticate patients with TBE and predict their outcome. Moreover, the need for human trials to help define the role of existing management options (e.g., mannitol, IVIg) and newly identified antiviral treatments is urgently required to improve outcomes.

## Figures and Tables

**Table 1:** Common symptom manifestations in both the first and second phases of disease of European TBE [63,64,65,66,67,68,69,70].

Symptoms	First Phase (%)	Second Phase (%)
Fever Headache Vomiting Pharyngitis Diarrhoea Myalgia Athralgia Ataxia Tremor Cranial nerve lesion Emotional instability Vertigo Seizures Hemiparesis	55–84 42–95.5 8–37.8 3–11 16–16.3 54–70.1 42.9–47	94–96.9 84–95.5 39–61 3 10 38.1 41 26–44 9–38 4–5.3 15 46.7 1 2.6

## Data Availability

No new data were created or analyzed in this study. Data sharing is not applicable to this article.
